# Reprogramming brain bioenergetics in depression: an integrative framework linking creatine, branched-chain amino acids, and exercise to neuroplasticity, cognitive function, and depression-related outcomes

**DOI:** 10.3389/fpsyt.2026.1830803

**Published:** 2026-08-04

**Authors:** Xiaoqin Xu, Li Liu

**Affiliations:** 1 Department of Physical Education, Hunan International Economics University, Changsha, Hunan, China; 2 Department of Physical Education, Hunan First Normal University, Changsha, Hunan, China

**Keywords:** branched-chain amino acids, cognitive function, creatine, depression, exercise, neuroplasticity, serotonin signaling

## Abstract

Depression represents a multifaceted neuropsychiatric disorder distinguished by disruptions in cerebral energy metabolism, neurotransmitter communication, neuroplasticity, and cognitive processes. An increasing body of literature indicates that integrative non-pharmacological interventions aimed at metabolic and neurochemical pathways may present promising adjunctive strategies for ameliorating depressive manifestations and concomitant cognitive impairments. This review explores the prospective combined effects of creatine supplementation, branched-chain amino acids (BCAAs), and physical exercise as a multimodal bioenergetic intervention for the management of depression. Creatine is pivotal in maintaining neuronal energy equilibrium via the phosphocreatine system, thereby facilitating mitochondrial functionality and adenosine triphosphate availability within neural cells. BCAAs may influence central fatigue and exercise performance through competitive inhibition of tryptophan transport across the blood-brain barrier. Importantly, this mechanism primarily reflects acute exercise-related serotonergic responses associated with central fatigue and should not be considered mechanistically equivalent to the chronic serotonergic dysfunction observed in major depressive disorder. Accordingly, within the context of depression, BCAAs are discussed as indirect modulators of mental health outcomes through their effects on fatigue perception, exercise tolerance, and adherence to physical activity, rather than as direct serotonergic antidepressant interventions. Concurrently, consistent engagement in physical exercise activates critical neuroplasticity-associated signaling pathways, which are instrumental in promoting hippocampal neurogenesis and enhancing stress resilience. Emerging empirical evidence derived from both experimental and clinical investigations suggests that the combined application of these interventions may exert complementary influences on brain bioenergetics, neuroplasticity, exercise capacity, and cognitive function. Collectively, this integrative paradigm highlights the potential of creatine supplementation, BCAAs, and physical exercise to support depression-related outcomes through distinct yet complementary mechanisms involving bioenergetic regulation, enhanced exercise capacity, neuroplastic adaptations, and improved cognitive and emotional functioning.

## 1 Introduction

Depression is defined not merely by enduring feelings of sadness and disinterest, but also by disruptions in cognitive processes, motivational states, sleep patterns, and the regulation of energy levels. While initial theoretical frameworks concerning depression predominantly emphasized monoamine deficiency, contemporary investigations suggest that the disorder emerges from intricate interactions involving neurotransmitter dysregulation, compromised neuroplasticity, neuroinflammatory processes, endocrine irregularities, and metabolic dysfunctions within neural networks (1, 2). Brain bioenergetics entails the mechanisms of how the brain synthesizes, stores, and utilizes energy to facilitate neuronal functioning, synaptic transmission, and cell maintenance (3). On the other hand, neuroplasticity is defined as the brain’s capacity to modify its structure and performance depending on experiences, injuries, or any interventions that involve synaptic strength, connections, and circuitry. When considering depression, it is evident that these two concepts have significant importance since metabolic energy disturbances and poor plasticity can be contributing factors to mood disorders (4).

Neuroimaging and molecular research reveal both structural and functional modifications in critical cerebral regions that are integral to emotional and cognitive processing, such as the hippocampus, prefrontal cortex, anterior cingulate cortex, and amygdala (5). Growing evidence posits that depression ought to be reconceptualized not solely as a disorder of neurotransmitter signaling but also as a condition characterized by dysregulated cerebral energy metabolism and diminished neuronal resilience (6, 7). Among the neurotransmitter systems that have been associated with depressive disorders, serotonin (5-hydroxytryptamine; 5-HT) remains one of the most rigorously examined due to its pivotal function in the regulation of mood, responsiveness to stress, emotional processing, and cognitive capabilities. Serotonergic neurons, which predominantly originate from the raphe nuclei, project extensively throughout the cerebral structure and modulate numerous neural circuits that are involved in affective regulation and executive functions. Disruptions in serotonergic transmission have been persistently documented in individuals diagnosed with depression, and the majority of first-line pharmacological interventions for depression operate by augmenting the availability of synaptic 5-HT (8). Nevertheless, the 5-HT hypothesis in isolation fails to comprehensively account for the heterogeneity observed in depressive disorders or the latency in therapeutic outcomes associated with antidepressants. In recent years, scholarly focus has increasingly been directed towards elucidating the ways in which serotonergic signaling interacts with other biological systems, such as metabolic pathways and neuroplasticity mechanisms (9).

One significant yet frequently overlooked dimension of 5-HT regulation pertains to the metabolic mechanisms that govern the availability of its precursor, tryptophan. The transport of tryptophan through the blood–brain barrier is facilitated by the large neutral amino acid transporter (LAT1), which is concurrently utilized by branched-chain amino acids (BCAAs) such as leucine, isoleucine, and valine. Due to the competitive nature of these amino acids for the same transport system, fluctuations in circulating concentrations of BCAAs can significantly affect the influx of tryptophan into the central nervous system and consequently influence the synthesis of 5-HT (10). This mechanism elucidates the intricate interconnection between amino acid metabolism, dietary consumption, and the regulation of central neurotransmitters. Moreover, variations in the plasma tryptophan/BCAAs ratio during episodes of physiological stress or physical exertion may modulate serotonergic function and subsequently impact fatigue, mood, and cognitive efficacy (11, 12). It is crucial to recognize that serotonergic responses elicited during extended periods of physical exercise should not be regarded as mechanistically analogous to the serotonergic impairments linked with major depressive disorder (MDD). Within the realm of exercise physiology, momentary increases in central serotonergic activity have been predominantly associated with the onset of central fatigue, which is significantly influenced by variations in the plasma free tryptophan/BCAA ratio and the ensuing competition for transport via LAT1 across the blood–brain barrier (13, 14). These acute physiological responses transpire over a time scale of minutes to hours during prolonged physical exertion and primarily engage neural circuits related to the perception of fatigue, particularly involving the lateral hypothalamus and amygdala. Conversely, serotonergic dysfunction pertinent to depression encompasses chronic modifications pertaining to the output of raphe nuclei, receptor sensitivity, synaptic plasticity, and regulatory mechanisms of limbic-cortical networks that develop over an extended duration of weeks to months (15, 16). Therefore, although BCAAs may modulate exercise-associated central fatigue through effects on tryptophan availability, this mechanism should not be directly extrapolated to explain antidepressant effects mediated through serotonergic pathways in MDD.

Importantly, this pathway is primarily relevant in the context of exercise physiology and central fatigue regulation, and its implications for clinical depression remain complex and not necessarily linear. These observations accentuate the necessity of incorporating metabolic and nutritional considerations when examining serotonergic dysfunction in the context of depression. Beyond the regulation of neurotransmitters, an increasing body of evidence indicates that depression is linked to perturbations in cerebral bioenergetics. Neurons, which are cells with a high demand for energy, rely predominantly on mitochondrial oxidative phosphorylation to sustain synaptic transmission, ion gradients, and cellular signaling pathways. Reports indicate that individuals suffering from depression and other affective disorders exhibit disruptions in mitochondrial functionality, ATP synthesis, and the systems that buffer phosphocreatine (7, 17). Investigations utilizing magnetic resonance spectroscopy have unveiled altered concentrations of high-energy phosphates and diminished ratios of creatine to phosphocreatine across various cerebral regions in patients diagnosed with depression, thereby implying compromised energy metabolism (18). Such deficiencies may undermine neuronal plasticity, hinder neurotransmitter biosynthesis, and disrupt synaptic signaling, ultimately exacerbating cognitive dysfunction and emotional instability.

Cognitive dysfunction has been recognized as a fundamental characteristic of depression, rather than merely an ancillary outcome of affective disturbances. Individuals diagnosed with depression frequently demonstrate deficits in attentional processes, executive functioning, memory retention, and the speed of information processing, which may persist even subsequent to the amelioration of mood-related symptoms (19). These cognitive impairments exert a substantial impact on occupational efficacy, interpersonal relationships, and overall life satisfaction. The neurobiological underpinnings of these cognitive deficits are complex and involve modifications in hippocampal neurogenesis, disruptions in synaptic plasticity, and the dysregulation of neurotrophic factors, including brain-derived neurotrophic factor (BDNF) (5). Notably, energy metabolism is integral to the facilitation of neuroplastic processes, indicating that metabolic interventions aimed at enhancing neuronal bioenergetics may possess the capacity to concurrently improve both mood and cognitive functions. Given the intricate and multifaceted pathophysiology underlying depression, there exists an increasing scholarly interest in complementary methodologies that address metabolic and lifestyle determinants in conjunction with traditional pharmacotherapeutic interventions. Nutritional supplementation and physical exercise have surfaced as promising non-pharmacological modalities that possess the capacity to modulate various biological pathways pertinent to the pathophysiology of depression. Exercise, in particular, has been consistently linked to antidepressant effects in both clinical and experimental research contexts (20). Regular engagement in physical activity has been shown to enhance hippocampal neurogenesis, elevate BDNF expression, improve mitochondrial functionality, and modulate monoaminergic neurotransmission (21, 22). Furthermore, exercise exerts influence on systemic metabolic processes, encompassing glucose utilization, lipid metabolism, and inflammatory signaling, which may contribute to its neuroprotective and antidepressant properties.

Creatine supplementation constitutes a further intervention that specifically targets cerebral bioenergetics. Creatine is integral to the phosphocreatine energy system, which serves to buffer adenosine triphosphate (ATP) availability during instances of elevated metabolic demand. Within the central nervous system, creatine is instrumental in the maintenance of cellular energy homeostasis, facilitating synaptic transmission, and safeguarding neurons against metabolic stress (23). Emerging research suggests that creatine supplementation may elevate brain phosphocreatine concentrations and enhance mitochondrial efficacy, thereby bolstering neuronal function and resilience (17, 24). BCAAs have garnered significant scholarly interest owing to their capacity to modulate neurotransmitter biosynthesis, energy metabolic processes, and intracellular signaling cascades. BCAAs engage in metabolic mechanisms that facilitate protein biosynthesis, optimize mitochondrial activity, and promote the activation of signaling pathways such as mechanistic target of rapamycin (mTOR), which is pivotal in regulating synaptic plasticity and neuronal development (25). Empirical investigations indicate that BCAAs may augment resilience to stress and influence BDNF signaling pathways within the hippocampal region (26). Although BCAAs compete with tryptophan for transport via LAT1, evidence supporting this mechanism derives predominantly from exercise physiology and central fatigue models rather than from studies conducted in patients with MDD. Consequently, BCAAs should not be considered direct modulators of the chronic serotonergic abnormalities observed in depression. Instead, their potential contribution to depression-related outcomes is more plausibly mediated through improvements in energy metabolism, exercise tolerance, and neuroplasticity-related signaling pathways. Furthermore, epidemiological research suggests that the dietary consumption of BCAAs may correlate with a diminished risk of experiencing depressive symptoms, thereby underscoring the potential significance of amino acid metabolism in the realm of mental health (27).

Thus, the purpose of this paper is to critically examine the available literature regarding the complementary roles of creatine supplementation, BCAAs, and physical exercise in modulating brain bioenergetics, neuroplasticity, exercise capacity, and depression-related outcomes. Particular emphasis is placed on distinguishing acute exercise-related serotonergic responses from chronic serotonergic dysfunction in depression in order to avoid mechanistic overgeneralization. In particular, it will be attempted to shed light on some of the metabolic and biochemical pathways involved in these treatments and the points where the metabolic and neurochemical systems intersect. Furthermore, the gaps in research in these areas will be identified for future experimental studies. In order to make explicit the rationale behind the choice of studies to include in the current literature review, an integrative literature review was performed concerning the interplay between brain bioenergetics, 5-HT metabolism, and multimodal treatments combining creatine, BCAAs, and physical activity.

## 2 Literature search strategy and study design

This study was conducted as a narrative review and does not follow a systematic review framework (e.g., PRISMA guidelines). The aim of this review was to provide an integrative overview of the current evidence regarding the potential effects of creatine supplementation, BCAAs, and exercise on depression-related outcomes, including neurobiological, cognitive, and behavioral mechanisms. A comprehensive literature search was performed using major scientific databases, including PubMed, Scopus, and Google Scholar. The search covered studies published from 1995 to December 2025 to ensure inclusion of both foundational and recent research relevant to the topic. The following keywords and their combinations were used during the search process: “creatine”, “creatine supplementation”, “branched-chain amino acids”, “BCAAs”, “exercise”, “physical activity”, “depression”, “major depressive disorder”, “serotonin”, “5-HT”, “cognition”, “neuroplasticity”, “BDNF”, and “brain energy metabolism”. Studies were selected based on their relevance to the scope of the review, focusing on research investigating the effects of creatine, BCAAs, and/or exercise on mood-related outcomes, cognitive function, or underlying neurobiological mechanisms in both human and animal models. Articles were included if they provided experimental, clinical, or mechanistic insights relevant to the topic. Non-English publications, editorials, commentaries, and studies not directly related to the central theme were excluded. Given the emerging and multidisciplinary nature of this field, evidence derived from clinically diagnosed MDD populations, animal models, healthy individuals, athletes, older adults, and exercise-based paradigms was considered where appropriate to provide a comprehensive conceptual framework. However, particular caution was exercised when extrapolating findings obtained from non-clinical populations to the pathophysiology or treatment of major depressive disorder. Moreover, mechanistic findings related to acute exercise physiology—particularly those involving serotonergic responses associated with central fatigue—were interpreted separately from evidence addressing chronic serotonergic dysfunction in depression in order to avoid mechanistic overgeneralization. Given the heterogeneity of the available literature and the exploratory nature of the topic, the evidence was synthesized narratively rather than quantitatively. Accordingly, the conclusions of this review should be interpreted as hypothesis-generating rather than definitive evidence of therapeutic efficacy.

## 3 Creatine combined with exercise in depression: effects on brain bioenergetics, neuroplasticity, and mood regulation

Although several studies support potential interactions among creatine, BCAAs, and exercise in neurobiological pathways associated with depression, much of the currently available evidence remains indirect. Many studies have been conducted in healthy athletes, older adults, sleep-deprived individuals, stroke survivors, or animal models rather than patients with MDD. Therefore, the evidence discussed in this review should be interpreted according to its clinical relevance to depression. Moreover, mechanistic evidence derived from exercise physiology, particularly studies examining acute serotonergic responses related to central fatigue, is discussed separately from evidence concerning chronic serotonergic dysfunction in MDD to avoid mechanistic overgeneralization (13, 15). To improve clarity, the studies were categorized as: (1) depression-related clinical evidence, (2) depression-related animal studies, (3) indirect mechanistic evidence, and (4) exercise performance- or fatigue-related evidence.

### 3.1 Creatine and brain energy metabolism

Creatine is a naturally occurring guanidine derivative, predominantly synthesized from the amino acids arginine, glycine, and methionine, and it assumes a pivotal role in the regulation of cellular energy homeostasis. In tissues characterized by significant and variable energy requirements, such as skeletal muscle and the cerebral cortex, creatine operates via the creatine–phosphocreatine (PCr) system, which functions as an immediate reservoir for the regeneration of ATP. The reversible enzymatic reaction facilitated by creatine kinase transfers a high-energy phosphate moiety from phosphocreatine to ADP, thus enabling prompt ATP resynthesis during phases of elevated metabolic demand (23, 28). Furthermore, this system also promotes intracellular energy transport from mitochondria, the sites of ATP synthesis, to the locations of ATP consumption within neurons and synapses. The cerebrum constitutes an organ with significant energetic demands, representing approximately 20% of the total body’s energy expenditure while comprising merely around 2% of the overall body mass. Consequently, the presence of an efficient energy metabolism is imperative for the preservation of neuronal excitability, the synthesis of neurotransmitters, the process of synaptic transmission, and the phenomenon of neuroplasticity. Disruptions in cellular bioenergetics have the potential to hinder these critical processes and are implicated in the pathophysiology of various neuropsychiatric conditions. An increasing body of evidence indicates that depressive disorders are correlated with modifications in mitochondrial functionality and the metabolism of high-energy phosphates. Neuroimaging investigations employing magnetic resonance spectroscopy have revealed diminished phosphocreatine concentrations and altered ATP dynamics within cerebral regions associated with mood regulation, particularly the prefrontal cortex and basal ganglia (17, 18). Such pathological abnormalities may undermine neuronal resilience and disrupt synaptic signaling, thereby exacerbating mood disturbances and cognitive impairments commonly observed in depressive disorders. Creatine supplementation has been posited as a viable intervention to augment cerebral bioenergetics and facilitate neuronal functionality. The enhancement of intracellular creatine availability has the potential to amplify phosphocreatine reservoirs and bolster ATP regeneration capacity during periods of metabolic stress. A plethora of experimental and clinical investigations have demonstrated that creatine supplementation elevates cerebral creatine concentrations and may enhance mitochondrial efficacy alongside neuronal energy buffering (24). Via these pathways, creatine may indirectly bolster the synthesis of neurotransmitters and the phenomenon of synaptic plasticity, both of which are fundamentally reliant on sufficient energy provision. Importantly, unlike the acute exercise-related serotonergic responses discussed elsewhere in this review, the putative antidepressant effects of creatine are thought to arise primarily through improvements in cerebral bioenergetics, mitochondrial function, and neuroplasticity rather than through direct modulation of serotonin availability (16, 29).

### 3.2 Combined effects of creatine and exercise on 5-HT signaling and depressive-like behaviors

Several studies reviewed in this section evaluate acute or subacute alterations in serotonergic markers following exercise, creatine supplementation, or physiological stressors. These findings primarily reflect adaptations related to exercise performance, central fatigue, and stress resilience and should not be interpreted as direct evidence for the correction of chronic serotonergic dysfunction in MDD. Consequently, serotonin-related outcomes reported in experimental studies are discussed herein as mechanistic indicators rather than definitive antidepressant pathways (13, 15). Chronic stress has the potential to elicit behaviors akin to depression and diminish neurogenesis within the hippocampus, specifically in the dentate gyrus (DG). Physical exercise is recognized for its capacity to augment the generation of new neurons, whereas creatine has been reported to exhibit antidepressant-like properties, although the underlying mechanisms remain ambiguous. Leem et al. aimed to elucidate whether treadmill exercise and/or creatine supplementation influence the Wnt/GSK3β/β-catenin signaling pathway in the DG under conditions of chronic mild stress. Mice were subjected to a regimen of 4 weeks involving stress, exercise, and/or creatine, followed by a series of behavioral assessments (tail suspension and forced swimming) and evaluations of neurogenesis indicators (Ki-67, DCX), GSK3β activity, and the presence of nuclear β-catenin. The behavioral impairments and reductions in neurogenesis induced by stress were attenuated through the implementation of exercise and creatine, concomitant with the activation of the Wnt/GSK3β/β-catenin pathway. The inhibition of Wnt signaling within the DG resulted in increased immobility, thereby affirming its significance. The combined intervention of exercise and creatine yielded greater neurogenic and antidepressant-like outcomes compared to either intervention alone (30) (**Table 1****).** Ahn et al. assessed the influence of a 4-week regimen of creatine monohydrate (CrM) supplementation and/or physical exercise on depressive behaviors and the expression of raphe 5-HT in murine models subjected to chronic mild stress. Male C57BL/6 mice were systematically allocated into five distinct cohorts: a non-stressed control group, a stressed control group, a group subjected to stress with creatine supplementation, a group undergoing stress with exercise, and a group experiencing stress with the combined intervention of creatine and exercise. Behavioral assessments, specifically the Tail Suspension Test and Forced Swimming Test, alongside immunohistochemical analyses for 5-HT in the dorsal and median raphe nuclei, were meticulously performed. Chronic stress was found to exacerbate depressive behaviors while concomitantly diminishing raphe 5-HT expression. Both creatine supplementation and exercise independently provided partial amelioration of depressive behaviors and facilitated the restoration of raphe 5-HT immunoreactivity. The application of combined creatine and exercise resulted in a more pronounced improvement in behavioral outcomes and serotonergic markers (31). However, because these findings were obtained in a murine chronic mild stress model, the observed changes in raphe 5-HT should be interpreted as preclinical mechanistic observations rather than direct evidence of normalization of serotonergic dysfunction in patients with MDD. A 24-week, double-blind, randomized, placebo-controlled investigation examined the impact of creatine supplementation, both in conjunction with and independently of strength training, on emotional and cognitive outcomes in elderly females. Participants were allocated into four distinct groups: placebo, creatine, placebo combined with strength training, and creatine augmented with strength training. Creatine was administered at a dosage of 4×5 g/day over a duration of 5 days, subsequently followed by a maintenance dose of 5 g/day. Cognitive functions (including memory, attention, and inhibitory control) alongside emotional states (measured via the Geriatric Depression Scale) were evaluated at baseline, as well as at 12 and 24 weeks, with assessments of muscle strength and dietary intake conducted at baseline and at the 24-week mark. The findings indicated that both strength training groups, irrespective of creatine supplementation, exhibited significant reductions in depression scores and enhancements in muscle strength compared to their non-trained counterparts. Isolated creatine supplementation did not yield any observable effects on emotional or cognitive outcomes, and neither intervention demonstrated any influence on cognitive performance or dietary intake. Strength training alone contributed to improvements in mood and strength, without any additional benefits conferred by creatine supplementation (32).

**Table 1:** Evidence regarding the effects of creatine supplementation combined with exercise on depression-related outcomes and brain 5-HT.

Participants/model	Intervention	Duration	Main outcomes	Findings	Evidence category	Study
Mice exposed to chronic mild stress	Treadmill exercise + creatine supplementation	4 weeks	Hippocampal neurogenesis, Wnt/GSK3β/β-catenin signaling, depressive-like behaviors (TST, FST)	Hippocampal neurogenesis ↑, Ki-67 & DCX-positive cells ↑, depressive-like behaviors ↓, Wnt/GSK3β/β-catenin signaling ↑	Depression-related animal model	(30)
Male C57BL/6 mice with chronic mild stress (n=48)	Creatine supplementation, exercise, or combined treatment	5 weeks	Depressive-like behaviors (TST, FST), serotonin (5-HT) expression in dorsal and median raphe nuclei	Depressive-like behaviors ↓, 5-HT neurons in DR & MnR ↑	Depression-related animal model	(31)
Older women in a randomized double-blind placebo-controlled trial	Creatine supplementation with or without strength training	24 weeks	Emotional status (Geriatric Depression Scale), cognitive performance, muscle strength	Emotional status ↑ with training, muscle strength ↑ with training, cognitive function ↔ with creatine, creatine alone ↔ emotional status	Indirect clinical/mechanistic evidence	(32)
Healthy adults, sleep deprivation (n=19)	Creatine supplementation (5 g x 4/day) + mild exercise	7 days	Cognitive and psychomotor performance, mood state, plasma catecholamines & cortisol	Cognitive & psychomotor performance ↑, Mood state ↑, Catecholamines & cortisol ↔	Indirect mechanistic evidence	(33)
Stroke survivors (Creatine n=5; Placebo n=3)	Progressive resistance training (PRT) ± creatine	10 weeks	Muscle strength, functional exercise capacity, cognition (MoCA), depression (CES-D), walking performance	Muscle strength ↑, Balance ↑, Cognition ↑, Depression ↓, Walking performance ↑ (creatine + PRT)	Indirect clinical evidence	(34)
Endurance-trained males (n=21)	Creatine (20 g/d) + glucose polymer vs. placebo	7 days	Brain 5-HT & DA modulators, thermophysiology, perceived effort, endurance performance	Rectal temp ↓, Heart rate ↓, Perceived fatigue ↓, Free-Trp ↓, Free-Trp:tyrosine ratio ↓, Endurance performance ↔ overall; ↑ in “responders”	Exercise/fatigue-related evidence	(35)

TST, Tail Suspension Test; FST, Forced Swimming Test; DG, Dentate Gyrus; DCX, Doublecortin; Ki-67, Marker of cell proliferation; Cr, Creatine; ST-CON, Stress Control; ST-Cr, Stress + Creatine; ST-Ex, Stress + Exercise; ST-Cr+Ex, Stress + Creatine + Exercise; MoCA, Montreal Cognitive Assessment; GAD-7, Generalized Anxiety Disorder Assessment-7; CES-D, Center for Epidemiological Studies Depression Scale; RMG, Random Movement Generation; Trp, Tryptophan; DA, Dopamine; 5-HT, Serotonin; Plc, Placebo; PRT, Progressive Resistance Training.

Sleep deprivation adversely affects cognitive and psychomotor faculties as well as emotional states, which can be attributed, in part, to diminished levels of creatine in the brain. McMorris et al. aimed to examine whether supplementation with creatine can alleviate these detrimental effects experienced during sleep deprivation accompanied by light physical exertion. A total of nineteen participants were randomly allocated to either a creatine group (5 g administered four times daily, n=10) or a placebo group (n=9) for a duration of seven days, employing a double-blind methodological framework. Evaluations of cognitive and psychomotor performance (including random movement generation, verbal and spatial memory recall, choice reaction time, and static balance) as well as emotional state were conducted at baseline and subsequently at 6, 12, and 24 hours of sleep deprivation. Additionally, plasma levels of catecholamines and cortisol were quantified at both 0 and 24 hours. Following the 24-hour period, the group receiving creatine exhibited significantly less pronounced declines in both performance and mood compared to the placebo group. Although there was an observable increase in norepinephrine and dopamine levels alongside a decrease in cortisol over time, no significant group differences were detected in plasma concentrations. Creatine supplementation was associated with better maintenance of cognitive performance and mood under sleep deprivation conditions (33). Butchart et al. examined the influences of progressive resistance training (PRT) in conjunction with or independent of creatine supplementation among individuals who have experienced a stroke. Participants were systematically assigned to either the creatine group (n=5) or the placebo group (n=3) and underwent a 10-week regimen of supervised PRT. Evaluations conducted prior to and subsequent to the intervention encompassed body composition, muscle thickness, muscle strength (1-repetition maximum), functional exercise capacity (6-minute walk test, Berg Balance Scale), cognitive function (Montreal Cognitive Assessment), and indicators of anxiety (GAD-7) and depression (CES-D). The implementation of PRT resulted in statistically significant improvements in muscle strength, balance, cognitive function, and depressive symptom scores, with greater walking improvements observed in the creatine group; however, the small sample size limits the robustness and generalizability of these findings (34). Hadjicharalambous et al. assessed the influence of creatine supplementation on the functionality of brain 5-HT and dopamine (DA), thermophysiological responses, as well as perceived exertion during extended physical activity in elevated temperatures. A cohort of twenty-one endurance-trained males participated in two constant-load cycling trials to the point of exhaustion at approximately 63% VO_2_max in an environment of 30 °C and 70% humidity, both prior to and subsequent to a 7-day regimen of creatine (20 g/day plus glucose) or placebo supplementation, employing a double-blind methodology. The administration of creatine resulted in a reduction of rectal temperature, heart rate, perceptions of leg fatigue, plasma free tryptophan levels, and the free-Trp:tyrosine ratio, yet did not elicit an enhancement in overall endurance performance among the entire participant group. When the subjects were categorized into “responders” and “non-responders” based on the degree of intramuscular creatine uptake, the responders exhibited greater performance improvements compared with non-responders (35). Importantly, this study was designed within the context of exercise physiology and central fatigue rather than depression. Therefore, the observed alterations in plasma free tryptophan availability and putative serotonergic activity should not be considered mechanistically equivalent to the chronic serotonergic disturbances characteristic of depressive disorders (13, 15).

The relationship among BCAAs, exercise, and serotonergic signaling is intricate and should be understood within a comprehensive metabolic framework. Research indicates that exercise can elevate the ratio of free tryptophan to BCAAs in the plasma, which may enhance tryptophan’s transportation across the blood-brain barrier (BBB), potentially boosting central 5-HT production. However, increased levels of circulating BCAAs can compete with tryptophan for transport through the LAT1, possibly diminishing the availability of tryptophan in the brain and, in theory, lessening serotonergic activity. Importantly, the serotonergic mechanisms associated with BCAA supplementation are most consistently established in exercise physiology and central fatigue paradigms, where transient changes in the plasma free tryptophan/BCAA ratio occur during prolonged exercise (13, 14). These acute exercise-induced serotonergic responses occur over minutes to hours and are primarily associated with fatigue perception and exercise performance. In contrast, serotonergic dysfunction in major depressive disorder involves chronic alterations in raphe nuclei output, receptor sensitivity, synaptic plasticity, and limbic-cortical circuitry that evolve over weeks to months (15, 16). Consequently, exercise-induced fluctuations in tryptophan transport across the blood–brain barrier should not be considered mechanistically equivalent to serotonergic abnormalities in MDD. Accordingly, within the present framework, BCAAs are primarily conceptualized as modulators of the central fatigue–exercise capacity axis through their effects on peripheral energy metabolism, skeletal muscle performance, attenuation of exercise-induced fatigue, and facilitation of sustained physical activity rather than as direct serotonergic antidepressant agents. Through supporting exercise adherence and recovery, BCAAs may indirectly contribute to improvements in mood, neuroplasticity, and brain bioenergetics.

Thus, the interaction between exercise and BCAAs should be interpreted as a context-dependent metabolic mechanism relevant primarily to exercise physiology rather than as a direct therapeutic mechanism targeting serotonergic dysfunction in MDD. Some works utilized only depressive-like behavioral tests but lacked any clinical validation of depression, reducing the potential translational value of the conclusions drawn. Furthermore, many mechanistic studies examining serotonin-related outcomes were conducted in healthy individuals, athletes, or experimental animal models. Therefore, caution is warranted when extrapolating these findings to clinically diagnosed MDD populations. Taken together, the mechanisms described in this section highlight the central role of brain bioenergetics, neuroplasticity, and context-dependent neurotransmitter regulation in depression-related outcomes.

## 4 Creatine combined with exercise and cognitive function

Before reviewing the available evidence, it should be noted that most studies examining the combined effects of creatine supplementation and exercise on cognition have been conducted in healthy, athletic, or aging populations rather than in individuals with clinically diagnosed depression. Accordingly, the findings discussed in this section should be interpreted primarily as mechanistic or performance-related evidence that may inform hypotheses regarding depression rather than as direct evidence of efficacy in MDD. Mabrey et al. (36) determined the impact of creatine nitrate and caffeine, both in isolation and in conjunction, on exercise performance and cognitive capabilities among resistance-trained male athletes. Utilizing a double-blind, randomized crossover methodology, twelve male participants were administered either 7 days of creatine nitrate (5 g/day), caffeine (400 mg/day), or a combination of both. The assessment of safety was conducted through blood analyses for enzyme levels and lipid profiles, followed by standardized resistance training (RT) exercises (bench press and leg press at 70% of one-repetition maximum) along with a Wingate anaerobic test. Cognitive abilities and cardiovascular responses were evaluated 45 minutes after the supplementation period. The simultaneous intake of creatine nitrate and caffeine yielded a statistically significant enhancement in cognitive performance, as evidenced by improved scores on the Stroop Word-Color Interference test, and was found to be more efficacious than caffeine administered alone. No statistically significant enhancements in exercise performance were recorded, and no adverse events were reported (36) (**Table 2**). Pires et al. examined the impact of a 28-day regimen of creatine supplementation on cognitive function subsequent to exhaustive physical exertion in female Muay Thai practitioners. Employing a double-blind, placebo-controlled, repeated-measures methodology, 26 athletes were randomly assigned to either receive CrM (3 g/day) or a placebo (maltodextrin) over the course of 28 days. Cognitive evaluations including assessments of visual and auditory reaction times, the Corsi block test, visual forward digit span, and the Erikson Flanker Task, were administered immediately post-exercise, as well as both prior to and following the supplementation period. The results indicated a significant temporal effect on auditory reaction time, alongside trends reflecting enhancements in visual reaction time, visual go/no-go reaction time, and Flanker task performance within the creatine cohort, whereas no notable changes were observed in the placebo group. These results imply that a 28-day period of creatine supplementation yields modest improvements in post-exercise cognitive performance*, thereby* supporting its potential ergogenic role for cognition (37). Ben Maaoui et al. demonstrated the ramifications of a seven-day CrM loading phase on variables such as sleep quality, cognitive functioning, physical performance, and recovery among physically active male subjects. Utilizing a randomized, double-blind, placebo-controlled crossover methodology, fourteen participants received either 20 g/day of CrM or a placebo while adhering to predetermined exercise regimens. The assessment of sleep quality was conducted through actigraphy in conjunction with subjective questionnaires; cognitive function was evaluated employing the digit cancellation test; physical performance was quantified utilizing a 5-meter shuttle run assessment; recovery and muscle soreness were monitored for a duration of up to 72 hours’ post-exercise. CrM supplementation yielded enhancements in subjective sleep quality, an earlier in-bed time, improved cognitive performance, as well as increases in both total and peak distances achieved during high-intensity intermittent exercise. Additionally, muscle soreness exhibited a reduction. Nevertheless, CrM did not exert any significant influence on objective sleep parameters, the rating of perceived exertion, fatigue index, performance decrement, or various other recovery indicators (38).

**Table 2:** Indirect mechanistic evidence regarding the effects of creatine supplementation combined with exercise on cognitive function.

Participants/model	Intervention	Duration	Main outcomes	Findings	Evidence category	Study
12 resistance-trained male athletes	Creatine nitrate (5 g/day), caffeine (400 mg/day), or combination	7 days	Cognitive function (Stroop test), exercise performance (bench press, leg press, Wingate test)	Co-ingestion ↑ cognitive function (Stroop test); ↔ exercise performance; no adverse events	Exercise/cognition-related evidence	(36)
26 female Muay Thai athletes	Creatine monohydrate 3 g/day vs. placebo	28 days	Cognitive performance immediately post-exercise (reaction time, Corsi block, digit span, Erikson Flanker)	Small improvements ↑ in cognitive performance in creatine group; ↔ placebo	Exercise/cognition-related evidence	(37)
14 physically active men	Creatine monohydrate loading 20 g/day vs. placebo	7 days	Sleep metrics (actigraphy, SSQ), cognitive function (digit cancellation test), physical performance (5 m shuttle run), recovery (PRS, DOMS)	↑ Sleep quality (SSQ); ↑ cognitive function (DCT); ↑ physical performance (total and best distance); ↓ muscle soreness; ↔ RPE, fatigue index, other recovery metrics	Exercise/cognition-related evidence	(38)
12 male participants (20–45 y) performing anaerobic exercise	Creatine monohydrate 5 g/day	20 days	Cognitive function (MoCA)	↑ Cognitive function after 20 days (P = 0.024)	Exercise/cognition-related evidence	(39)
49 healthy older men (73 ± 6 y)	Multi-ingredient supplement: n-3 PUFA 1500 mg, whey protein 30 g, creatine 2.5 g, vitamin D 500 IU, calcium 400 mg; ± RET + HIIT	20 weeks (Phase 1: supplementation; Phase 2: supplementation + RET + HIIT)	Cognitive function (MoCA), memory (RAVLT), reaction time, n-3 index	Phase 2: ↑ MOCA, ↑ word recall, ↑ reaction time; within-group improvements in composite cognitive score in SUPP group; ↑ n-3 index; ↓ ARA: EPA ratio	Indirect mechanistic/clinical evidence	(40)
40 male adolescent basketball players (13–14 y)	Acute creatine monohydrate 0.3 g/kg/day for 5 days + 0.1 g/kg pretest	5 days (with 4-week washout)	Technical performance under cognitive-motor dual-task (dribbling, passing, shooting), RPE, heart rate	↑ Performance under CMDT (dribbling, passing, shooting); ↑ Performance under ST (dribbling, shooting); ↓ dual-task cost (dribbling, passing); ↑ subtraction task accuracy (shooting); ↓ HR and RPE	Exercise/cognition-related evidence	(41)

TST, Tail Suspension Test; FST, Forced Swimming Test; DG, Dentate Gyrus; DCX, Doublecortin; 5-HT, Serotonin; DR, Dorsal Raphe; MnR, Median Raphe; MoCA, Montreal Cognitive Assessment; BBS, Berg Balance Scale; CES-D, Center for Epidemiological Studies Depression Scale; PRT, Progressive Resistance Training; RMG, Random Movement Generation; RPE, Rating of Perceived Exertion; CMDT, Cognitive-Motor Dual Task; ST, Single Task.

Hariyanto et al. explored the impact of CrM supplementation on cognitive functionality in male individuals engaged in anaerobic exercises. A cohort of twelve male subjects, aged between 20 and 45 years, participated in anaerobic training regimens while ingesting a daily dosage of 5 g of CrM over a span of 20 days. Cognitive capabilities were evaluated on day 0 and day 21 utilizing the XpressO MoCA assessment tool. The participants exhibited an average age of 31.2 ± 7.6 years, a weight of 80.9 ± 14.6 kg, a height of 170.6 ± 5.1 cm, and a body mass index (BMI) of 27.8 kg/m². The findings revealed a statistically significant enhancement in cognitive performance following the 20-day creatine supplementation period, with scores escalating from 32.33 ± 9.7 at baseline to 46.75 ± 9.8 subsequent to the intervention (P = 0.024). These findings suggest a potential association between creatine supplementation and improved cognitive test performance under anaerobic exercise conditions, although the small sample size limits generalizability (39). Bell et al. determined the impact of multi-ingredient supplementation, both in conjunction with and independent of physical exercise, on cognitive functioning among healthy elderly males. A total of forty-nine sedentary male participants (mean age 73 ± 6 years; mean BMI 28.5 ± 3.6 kg/m²) were randomly assigned to receive either a supplement comprising n-3 polyunsaturated fatty acids (PUFAs), whey protein, creatine, vitamin D, and calcium (designated as SUPP) or a control beverage (denoted as CON) over a duration of 20 weeks. The initial phase concentrated solely on supplementation, while the subsequent phase incorporated an additional 12 weeks of RT combined with high-intensity interval training (HIIT). Cognitive evaluations encompassed the Montreal Cognitive Assessment, Rey Auditory Verbal Learning Test, and measurements of executive function reaction times. No discernible cognitive alterations were detected during the first phase; conversely, the second phase yielded notable enhancements in MOCA scores, word recall, and reaction time. Within-group analyses suggested that cognitive improvements were observed only in the supplemented group under combined intervention conditions, indicating a possible synergistic effect between exercise and nutritional supplementation, although causality cannot be established (40). Wu et al. explored the ramifications of acute creatine supplementation on the technical performance of adolescent basketball players in both single-task (ST) and cognitive-motor dual-task (CMDT) scenarios. A cohort of forty male athletes aged 13–14 years participated in a randomized, counterbalanced crossover study, wherein they were administered either CrM (0.3 g/kg/day for a duration of five days, alongside 0.1 g/kg prior to testing) or a placebo, with a four-week washout period implemented. The CMDT conditions necessitated that players engage in continuous subtraction while simultaneously executing dribbling, passing, and shooting maneuvers. Performance metrics, cognitive accuracy, heart rate, and perceived exertion levels were systematically assessed. The administration of creatine resulted in a significant enhancement in dribbling, passing, and shooting performance under CMDT conditions, as well as improvements in dribbling and shooting performance under ST circumstances. The dual-task cost was notably reduced in relation to dribbling and passing activities. Accuracy in subtraction tasks showed improvement during shooting activities. Furthermore, creatine supplementation was associated with a reduction in heart rate and ratings of perceived exertion across all tasks. These findings suggest that creatine may enhance cognitive-motor efficiency under high-demand physical conditions rather than exerting a direct cognitive enhancement effect (41).

A number of methodological issues must be taken into account when reviewing the literature on exercise-induced neuroplasticity and cognition. While the bulk of existing research has been conducted with humans, numerous studies have utilized extremely small sample sizes, narrow participant pools, and sports- or age-related groups of participants, which decreases the generalizability of the findings. Also, some of the investigations involved crossover or pre/post studies, which are effective at measuring short-term effects but still are susceptible to carryover and learning effects, among other biases. Moreover, in some cases, the researchers measured cognition via task-based performance measures, not actual biomarkers of neuroplasticity. Finally, since the majority of supplement studies employed formulations containing more than one ingredient, it was challenging to establish the exact role of creatine in their cognitive effects. Importantly, most studies involve healthy, athletic, or older non-clinical populations; therefore, observed effects should be interpreted as performance-related or mechanistic findings rather than clinical effects in MDD.

In conclusion, the evidence presented in this section suggests that creatine may influence cognitive performance and neurophysiological processes related to brain energy metabolism and stress resilience, particularly under conditions of physical or metabolic demand. Through its role in cellular energy buffering, creatine may contribute to maintaining neuronal energy homeostasis, which is relevant to brain function under stress. However, these findings are derived predominantly from studies involving healthy athletes, physically active individuals, older adults, or other non-clinical populations. Therefore, the observed cognitive benefits should primarily be interpreted as performance-related or mechanistic effects rather than direct evidence of therapeutic efficacy in MDD. However, it is critical to emphasize that the evidence presented in this section is largely derived from non-clinical populations, and therefore its implications for depression are indirect and mechanistic rather than clinically confirmatory. It is important to acknowledge that much of the evidence discussed in this section derives from studies conducted in healthy individuals, athletic populations, older adults, or general experimental models rather than in clinically diagnosed populations with MDD. Although these findings provide valuable mechanistic insights into how exercise, supplementation, and metabolic modulation influence cognition, cognitive enhancement observed in healthy states cannot be directly equated with cognitive remediation in pathological conditions such as depression. The pathophysiology of cognitive impairment in MDD involves additional neurobiological processes, including altered affective processing, inflammatory signaling, chronic stress-related neural remodeling, and disturbances in limbic-cortical networks, which are not fully captured in healthy populations. Consequently, the relevance of these findings to depressive disorders remains largely inferential, and direct clinical evidence investigating the combined effects of creatine and exercise on cognition in patients with MDD is currently limited. Future well-designed clinical studies specifically targeting depressive populations are required to determine whether the cognitive and neurobiological benefits observed in non-clinical cohorts translate into meaningful therapeutic outcomes in MDD.

## 5 BCAA combined with exercise in depression: effects on brain 5-HT

### 5.1 BCAAs and tryptophan competition at the blood–brain barrier

BCAAs are classified as essential amino acids that assume pivotal functions in protein biosynthesis, metabolic homeostasis, and cellular signaling pathways. Beyond their peripheral metabolic roles, BCAAs exert influence over central nervous system physiology by modulating the transport of amino acids across the BBB. The translocation of large neutral amino acids (LNAAs) into the cerebral environment predominantly occurs via the L-type amino acid transporter 1 (LAT1), which facilitates the transport of substrates such as tryptophan, phenylalanine, tyrosine, and BCAAs. Given that these amino acids utilize the same transport mechanism, they engage in competitive interactions for access to the brain, rendering their relative plasma concentrations a critical factor in determining cerebral amino acid availability (42, 43). Tryptophan serves as the primary precursor for 5-HT, a neurotransmitter that is fundamentally significant for the regulation of mood, emotional processing, and cognitive functions. However, it is important to recognize that serotonergic processes involved in exercise physiology are not mechanistically identical to those implicated in MDD. Whereas exercise-related serotonergic responses primarily reflect acute fluctuations in precursor availability and neurotransmitter synthesis, depression-associated serotonergic dysfunction involves chronic alterations in raphe nuclei activity, receptor sensitivity, synaptic plasticity, and limbic-cortical network function. The rate at which 5-HT is synthesized within the brain is profoundly affected by the quantity of tryptophan that is transported across the BBB. Thus, the proportion of plasma tryptophan relative to competing LNAAs is deemed a pivotal element in modulating serotonergic activity. Elevated levels of circulating BCAAs may hinder the transport of tryptophan into the brain by intensifying competition for the LAT1 transporter, consequently restricting central 5-HT synthesis in the context of acute exercise physiology and central fatigue regulation (10). Importantly, this mechanism reflects transient alterations in precursor availability and should not be interpreted as evidence that BCAA supplementation directly suppresses or normalizes serotonergic dysfunction in clinical depression. In contrast, an increase in the tryptophan/BCAA ratio promotes enhanced transport of tryptophan into the brain and may potentiate serotonergic neurotransmission. Importantly, this competition mechanism describes amino acid transport dynamics at the BBB and should not be interpreted as evidence that BCAA supplementation has direct antidepressant effects in clinical depression. During extended or vigorous exercise, skeletal muscle progressively utilizes BCAAs as an energy source, resulting in a decrease in circulating BCAA concentrations. Concurrently, physical exertion promotes lipolysis, increasing plasma free fatty acid concentrations and displacing tryptophan from albumin, thereby increasing free tryptophan availability for brain uptake. The combined effect of these metabolic changes increases the plasma free tryptophan/BCAA ratio, facilitating tryptophan transport across the BBB and potentially enhancing central 5-HT synthesis within serotonergic neurons (12, 13). Importantly, these exercise-induced alterations occur over a time scale of minutes to hours and are primarily linked to fatigue perception, exercise performance, and central fatigue mechanisms. In contrast, serotonergic dysfunction in MDD represents a chronic neurobiological disturbance involving long-term alterations in serotonergic circuitry that cannot be explained solely by acute changes in BBB amino acid transport. Therefore, the effects of BCAAs on tryptophan transport should be viewed primarily as mechanisms relevant to exercise capacity and fatigue regulation rather than direct therapeutic mechanisms targeting serotonergic dysfunction in depression.

### 5.2 Regulation of 5-HT and central fatigue

5-HT functions as a pivotal neuromodulator that plays a significant role in the modulation of emotional states, motivational processes, levels of arousal, and the experience of fatigue. Within the realm of exercise physiology, serotonergic dynamics have been intricately linked to the manifestation of central fatigue, a phenomenon that is characterized by a diminished motor drive, heightened perception of exertion, and a decline in both physical and cognitive performance during extended periods of exercise. It is important to emphasize that central fatigue is a construct originating from exercise physiology and refers specifically to performance-related declines in motor output, which is conceptually and biologically distinct from clinical depressive symptoms. The central fatigue hypothesis posits that the augmentation of 5-HT synthesis and release within specific cerebral regions during prolonged exercise contributes to feelings of exhaustion and reduced motivation by modifying neural activity within motor and limbic pathways (44). The biosynthesis of 5-HT in the central nervous system is predominantly contingent upon the concentration of its precursor, tryptophan. Given that tryptophan contends with BCAAs for translocation across the BBB, variations in the plasma tryptophan-to-BCAAs ratio can profoundly affect serotonergic neurotransmission. Although increased serotonergic activity during prolonged exercise is associated with fatigue perception in performance settings, this physiological phenomenon should not be interpreted as evidence of serotonergic dysfunction relevant to MDD. In the context of sustained physical exertion, the cumulative effects of BCAA catabolism by skeletal muscle coupled with elevated plasma free fatty acid levels increase the ratio of free tryptophan available for brain uptake. This alteration facilitates 5-HT synthesis within serotonergic neurons, particularly within brain regions implicated in fatigue perception and motivational regulation during exercise (13). Increased serotonergic activity under these conditions has been associated with exercise-induced central fatigue, manifested by heightened perceptions of effort and reduced motor performance. Importantly, these acute serotonergic responses occur over a time scale of minutes to hours and primarily reflect physiological adaptations to prolonged exercise. In contrast, serotonergic dysfunction in major depressive disorder involves chronic disturbances in raphe nuclei output, receptor sensitivity, synaptic plasticity, and limbic-cortical circuitry that evolve over weeks to months. Therefore, exercise-induced increases in central 5-HT synthesis should not be interpreted as mechanistically equivalent to the serotonergic abnormalities underlying MDD. Accordingly, within the present framework, modulation of the tryptophan/BCAA ratio is considered primarily relevant to the central fatigue–exercise performance axis rather than a direct therapeutic mechanism targeting serotonergic dysfunction in depression. Any potential benefits of BCAA supplementation for mood are therefore likely to occur indirectly through improved exercise tolerance, adherence to physical activity, and subsequent neuroplastic adaptations rather than through direct enhancement of serotonergic antidepressant signaling.

### 5.3 Effects of BCAAs and exercise on depressive symptoms

A pilot randomized, double-blind, placebo-controlled investigation investigated the effects of BCAAs in conjunction with exercise on physical performance, fatigue, and quality of life among older adults. A total of twenty participants (mean age 70.5 years; BMI 35 ± 2 kg/m²) were allocated to engage in eight weeks of moderate aerobic and RT while receiving either BCAAs (100 mg/kg/day) or a placebo. Physical performance was evaluated through handgrip strength, chair stands, gait speed, VO_2_max, and a 400 m walk; psychological well-being was assessed using the CES-D, Fatigue Assessment Scale, Insomnia Severity Index, and visual analog scales for pain, fatigue, and quality of life. The combination of exercise and BCAAs yielded significant enhancements in strength, mobility, and endurance relative to exercise combined with placebo, while concurrently diminishing fatigue (−45% vs. +92%) and symptoms of depression (−29% vs. +5%). However, due to the small sample size and strong confounding effect of exercise, the observed reduction in depressive symptoms cannot be attributed specifically to BCAA supplementation (45) (**Table 3****).** Importantly, improvements in depressive symptom scores observed in exercise-based interventions should not be interpreted as evidence that BCAA-induced alterations in tryptophan transport directly normalize serotonergic dysfunction in depression. Rather, any potential mood-related benefits are more plausibly explained by improved exercise capacity, reduced fatigue perception, and enhanced adherence to physical activity, all of which are independently associated with favorable neuroplastic and psychological outcomes. Caldo-Silva et al. evaluated the effects of BCAAs supplementation and multicomponent exercise (ME) on frailty, functional capacity, and mood in older adults. Thirty-five participants (mean age 83 ± 3 y) from residential care homes underwent a 40-week intervention including 16 weeks of exercise and/or BCAAs supplementation, an 8-week washout, and 16 weeks of retraining. Experimental groups included ME+BCAAs, ME alone, BCAAs alone, and control. Functional capacity was assessed via the Short Physical Performance Battery, frailty via Fried’s phenotype, mood via Geriatric Depression Scale and POMS, cognition via MMSE, and salivary testosterone measured anabolic response. Exercise improved functional capacity and prevented frailty progression, while BCAAs alone had limited impact but may mitigate detraining effects. Salivary testosterone correlated with handgrip strength. No significant changes were observed in mood, cognition, or depression, indicating exercise is the primary driver of functional improvements in frail older adults. Importantly, no significant improvements were observed in mood or depression-related outcomes, suggesting that BCAAs do not consistently exert direct antidepressant effects in aging populations (46). Hsu et al. assessed the impact of a beverage comprising BCAAs, arginine, and carbohydrates (designated as BCAAs drink) on both biochemical and psychological recovery following exhaustive physical exertion in a cohort of 14 healthy male subjects. The participants engaged in two experimental sessions utilizing a crossover design, during which they ingested either the BCAAs drink (BA) or a placebo (PL). Blood specimens were obtained prior to the exercise intervention and at several intervals extending up to 24 hours’ post-exercise. Although levels of lactate, ammonia, creatine kinase, and glycerol exhibited no significant variation between the trials, glucose and insulin concentrations were markedly elevated in the BA trial at 40–60 minutes, while the testosterone-to-cortisol ratio demonstrated a significant increase at 120 minutes, indicating an augmented anabolic response. Psychological evaluations conducted through the Profile of Mood States indicated that feelings of fatigue escalated immediately following exercise in both experimental conditions; however, only the BA trial exhibited a statistically significant diminution in fatigue by 120 minutes. Other psychological parameters, including vigor, anger, confusion, and depression, remained predominantly stable, thereby suggesting that the BCAAs Drink facilitates early recovery and mitigates post-exercise fatigue. The observed reductions in fatigue reflect post-exercise metabolic recovery rather than modulation of depressive pathology (47).

**Table 3:** Evidence regarding the effects of BCAA supplementation combined with exercise on depression-related outcomes and brain 5-HT.

Participants/model	Intervention	Duration	Main outcomes	Findings	Evidence category	Study
20 older adults (63% female; BMI 35 ± 2 kg/m²; age 70.5 ± 1.2 y)	Exercise + BCAAs (100 mg/kg/d) vs. Exercise + Placebo	8 weeks	Physical function (handgrip, chair stand, 400 m walk, gait speed, VO_2_max), fatigue, depressive symptoms, quality of life	Exercise + BCAA improved handgrip strength ↑, chair stands ↑, 400 m walk time ↓; reduced fatigue ↓ (-45% vs +92%) and depressive symptoms ↓ (-29% vs +5%); ISI ↓30%, FAS ↓21%, VAS QoL ↑16%	Indirect clinical evidence related to depressive symptoms	(45)
35 frail older adults (83 ± 3 y)	Multicomponent exercise (ME) ± BCAA supplementation; 16-week exercise + 8-week washout + 16-week retraining	40 weeks	Functional capacity (SPPB), frailty (Fried’s phenotype), mood (GDS, POMS), cognition (MMSE), salivary testosterone (ST)	Exercise improved functional capacity ↑ and prevented frailty ↑ in CG; BCAA alone no effect on functional fitness; BCAA + exercise may reduce detraining effects; no effect on mood, cognition, depression	Indirect clinical evidence	(46)
14 healthy males	BCAA + arginine + carbohydrate drink vs. Placebo	Single session, recovery 24 h	Biochemical response (glucose, insulin, testosterone/cortisol), psychological condition (POMS)	BA trial ↑ glucose & insulin at 40–60 min; ↑ T/C ratio at 120 min; fatigue ↓ at 120 min recovery; vigor recovered; anger, confusion, depression unchanged; anabolic response supported by BCAA drink	Exercise/fatigue-related evidence	(47)
Male C57BL/6 mice	BCAA (leucine, isoleucine, valine) ± voluntary exercise; high protein diet (HPD)	10 days CSDS; 2-week diet intervention	Stress resilience (social interaction, elevated plus maze, open field), hippocampal BDNF/TRKB signaling	BCAA ↑ resilience, rescued social avoidance ↑; ↑ hippocampal BDNF & TRKB activation; TRKB inhibition abolished effect; voluntary exercise and HPD similarly ↑ resilience; combined BCAA + exercise no synergistic effect	Depression-related animal model	(26)
Sedentary & exercising rats	Oral BCAA ± tyrosine co-administration	Single session	Brain serotonin (5-HT) & catecholamine synthesis, tryptophan & tyrosine concentrations	BCAA ↓ brain tryptophan ↓, tyrosine ↓; ↓ serotonin & catecholamine synthesis; tyrosine co-administration prevented catecholamine reduction; may explain inconsistent effects on physical performance	Indirect mechanistic evidence	(48)
10 endurance runners (7 M, 3 F)	BCAA (0.17 g·kg^-^¹), arginine (0.05 g·kg^-^¹), citrulline (0.05 g·kg^-^¹) vs placebo	2 consecutive days	Endurance performance (5000 m, 10000 m), RPE, tryptophan/BCAA ratio, NH_3_, urea	AA trial ↑ performance 5000 m & 10000 m; ↓ tryptophan/BCAA ratio; NH_3_ similar; ↑ urea; improved performance likely via cerebral serotonin inhibition & hyperammonemia prevention	Exercise/fatigue-related evidence	(49)
Exercising rats	Voluntary intake of BCAA + L-arginine + L-glutamine solution; BCAA-fortified diet (2% wt:wt)	Multiple days during circadian dark (active) period	Exercise preference, physical activity volume, plasma BCAA/tryptophan ratio, brain 5-HT levels (lateral hypothalamus & amygdala)	Rats voluntarily preferred BCAA solution during intense exercise ↑; intake ↑ physical activity from day 4 ↑; BCAA solution ↑ plasma BCAA/Trp ratio ↑; ↓ 5-HT release in lateral hypothalamus & amygdala ↓; suggests ergogenic and central fatigue benefits	Indirect mechanistic evidence	(50)
Free-running rats	Free access to running wheel + BCAA + glutamine + arginine solution	Short-term fluid access during dark period + treadmill running	Exercise preference, lateral hypothalamus & amygdala 5-HT release, plasma amino acids	Dark-period running distance positively correlated with BCAA solution preference ↑; BCAA solution ↑ plasma BCAA/Trp ratio ↑; ↓ serotonin release in lateral hypothalamus ↓; no change in amygdala; lateral hypothalamus critical for BCAA central effects; supports ergogenic benefit	Indirect mechanistic evidence	(51)

BCAA, branched-chain amino acids; ME, multicomponent exercise; CG, control group; CES-D, Center for Epidemiologic Studies Depression Scale; FAS, Fatigue Assessment Scale; ISI, Insomnia Severity Index; VAS, visual analog scale; GDS, Geriatric Depression Scale; POMS, Profile of Mood States; MMSE, Mini-Mental State Examination; SPPB, Short Physical Performance Battery; ST, standard diet; HPD, high protein diet; CSDS, chronic social defeat stress; BDNF, brain-derived neurotrophic factor; TRKB, tropomyosin receptor kinase B; AA, BCAA + arginine + citrulline; PL, placebo; 5-HT, 5-hydroxytryptamine.

Nasrallah et al. examined the impact of BCAAs, physical exercise, and high-protein diets (HPD) on stress resilience and depression-like behaviors in male C57BL/6 mice exposed to chronic social defeat stress. Mice administered BCAAs exhibited diminished social avoidance, augmented expression of BDNF in the hippocampus, and heightened activation of TRKB. The blockade of TRKB signaling negated these protective effects, thereby underscoring the critical significance of BDNF/TRKB signaling pathways. Furthermore, BCAAs were found to activate the PGC1α/FNDC5 pathway, akin to the effects of voluntary exercise; however, the combination of BCAAs and exercise did not produce advantages. The HPD replicated the effects of BCAAs, resulting in a substantial enhancement of stress resilience (85.7% compared to 26% in the standard diet), fostering social interaction, and elevating the expression of hippocampal BDNF mRNA, independently of variations in body weight or locomotor activity. Although BDNF/TRKB signaling alterations were observed in animal models, these findings remain preclinical and require validation in human depressive populations before translational interpretation (26). An investigation scrutinized the impact of BCAAs on the synthesis of neurotransmitters within the brains of both sedentary and exercising rat models. Within exercise physiology, prolonged physical activity may increase central serotonergic activity in brain regions implicated in fatigue perception. Under these conditions, BCAAs can reduce brain tryptophan availability through competition at the LAT1 transporter, thereby attenuating exercise-induced increases in central 5-HT synthesis. Importantly, these observations relate specifically to acute central fatigue mechanisms and should not be interpreted as evidence that reducing central 5-HT is beneficial in depression. Nonetheless, BCAAs concurrently impede the uptake of tyrosine, thereby attenuating catecholamine synthesis, which is vital for the augmentation of physical performance capabilities. One hour subsequent to the administration of BCAAs via oral route, the subjects exhibited diminished concentrations of tryptophan and tyrosine in the brain alongside a reduction in both 5-HT and catecholamine synthesis. The concomitant administration of tyrosine alongside BCAAs mitigated the decrease in tyrosine and catecholamine synthesis, yet did not influence 5-HT synthesis. Comprehensive essential amino acid mixtures demonstrated disparate effects on neurotransmitter synthesis (48). Cheng et al. assessed the impact of a supplementation of BCAAs, arginine, and citrulline on endurance performance over a span of two consecutive days involving ten runners. Subjects ingested 0.17 g/kg BCAAs, 0.05 g/kg arginine, and 0.05 g/kg citrulline (AA trial) or a placebo (PL trial) in a randomized crossover framework, executing a 5000 m time trial on the first day and a 10,000 m trial on the second day. The AA trial demonstrated a statistically significant enhancement in performance across both trials in comparison to the placebo, without influencing perceived exertion levels. From a biochemical perspective, the AA trial resulted in a reduction of the tryptophan/BCAAs ratio, stabilization of ammonia concentrations, and an elevation in urea levels. These findings imply that BCAAs supplementation may attenuate cerebral 5-HT synthesis, thereby postponing the onset of central fatigue, while arginine and citrulline mitigate hyperammonemia through augmented urea synthesis. These findings support a role of BCAAs in delaying exercise-induced central fatigue; however, this should not be interpreted as evidence of antidepressant efficacy (49).

Smriga et al. examined the ramifications of voluntary BCAAs consumption, in conjunction with L-arginine and L-glutamine, on exercise behavior and central fatigue in rodent models. In periods of strenuous physical exertion, the subjects exhibited a marked preference for a BCAAs-enhanced solution over standard water, with consumption levels demonstrating a positive correlation to the timing and volume of exercise. The provision of a diet enriched with BCAAs (2% wt:wt) resulted in a significant escalation in voluntary physical activity commencing on the fourth day of observation. Neurobehavioral assessments disclosed that BCAAs supplementation led to an increase in the plasma BCAAs/tryptophan ratio while concurrently diminishing the release of 5-hydroxytryptamine (5-HT) in the lateral hypothalamus and amygdala post-exercise. These observations indicate that BCAAs may alleviate exercise-induced central fatigue through modulation of serotonergic signaling within specific brain regions, notably the lateral hypothalamus and amygdala. Importantly, these regions and mechanisms are primarily involved in acute fatigue regulation during exercise and should not be considered mechanistically equivalent to the chronic serotonergic abnormalities observed in MDD. The voluntary inclination towards BCAAs solutions, coupled with their influence on neurotransmitter equilibrium, suggests an ergogenic advantage, establishing a connection between central 5-HT regulation and enhanced motivation and endurance during exercise in conditioned rats (50). Smriga et al. investigated the impact of a BCAAs-enriched solution (BCAAs in conjunction with glutamine and arginine) on voluntary physical activity and central 5-HT signaling pathways in a rat model. Rodents granted unrestricted access to running wheels exhibited a significant positive correlation between the distance covered during the dark phase and their preference for the BCAAs solution. Neurochemical assessments indicated that the intake of the BCAAs solution resulted in a diminished release of 5-HT within the lateral hypothalamus, while there were no changes observed in the amygdala or raphe nuclei. In rats subjected to treadmill training, administration of the BCAAs solution prior to exercise resulted in an increase in the plasma BCAAs/tryptophan ratio and a subsequent decrease in 5-HT release from the lateral hypothalamus during and for 80 minutes following running. These results indicated that BCAAs supplementation influences serotonergic activity within the lateral hypothalamus, potentially alleviating exercise-induced central fatigue and augmenting motivation for voluntary physical activity. However, the lateral hypothalamic serotonergic responses observed in this animal model represent acute exercise-related neurochemical adaptations and should not be extrapolated to serotonergic dysfunction in depressive disorders. The observed preference for the BCAAs solution, along with its neurochemical implications, suggests an ergogenic advantage, emphasizing the lateral hypothalamus as a critical cerebral region through which BCAAs exerts its central effects during endurance-related activities. These results primarily reflect motivational and neurochemical adaptations in animal exercise behavior models rather than clinical relevance to depression (51).

The evidence presented in this section remains limited and heterogeneous, consisting predominantly of animal studies, small human trials, and exercise-based experimental models. In many human investigations, BCAAs were administered together with other amino acids or nutritional supplements, making it difficult to isolate the specific contribution of BCAAs. Furthermore, most available studies evaluated acute exercise responses or short-term interventions rather than long-term clinical outcomes in individuals with MDD. Methodological limitations, including small sample sizes and inconsistent reporting of blinding procedures, allocation concealment, and statistical power, further restrict interpretation. Importantly, the current body of evidence primarily supports a role for BCAAs in peripheral metabolism, exercise performance, and central fatigue regulation rather than providing robust clinical evidence for antidepressant efficacy in MDD. The serotonergic effects of BCAAs described in exercise studies largely reflect acute alterations in tryptophan availability and neurotransmitter synthesis occurring over minutes to hours, particularly within brain regions involved in fatigue perception such as the lateral hypothalamus and amygdala. In contrast, serotonergic dysfunction in depression involves chronic disturbances in raphe nuclei signaling, receptor regulation, and limbic-cortical circuitry over substantially longer time scales. Therefore, within the conceptual framework proposed in this review, BCAAs should primarily be viewed as modulators of exercise capacity and fatigue resistance. Any potential influence on depressive outcomes is likely indirect and mediated through improved engagement in physical activity and subsequent neuroplastic adaptations rather than through direct modulation of serotonergic antidepressant pathways.

## 6 BCAAs combined with exercise and cognitive function

Before discussing the available evidence, it is important to acknowledge that most studies investigating the combined effects of BCAAs and exercise on cognition have been conducted in healthy individuals, athletes, older adults, or animal models rather than in patients with clinically diagnosed MDD. Consequently, the findings presented in this section should be interpreted primarily as mechanistic or performance-related evidence rather than direct evidence of therapeutic efficacy for cognitive dysfunction in depression. An investigation scrutinized the impact of the BioSteel High Performance Sports Drink (B-HPSD), which comprises BCAAs and vitamin B-6, on the performance of multiple sprint exercise (MSE) among eleven seasoned cyclists. The subjects engaged in two counterbalanced trials, during which they ingested either B-HPSD or a placebo, subsequently executing five maximal 1 km sprints with a two-minute interval of active recovery between each sprint. Parameters such as power output, heart rate, ratings of perceived exertion, blood lactate levels, glucose concentrations, and cognitive functioning were meticulously evaluated. Despite the observed decline in power output across the sprints and the concomitant increase in lactate levels, heart rate, and perceived exertion ratings, no discernible differences in performance, perceived exertion, or cognitive function were identified between the B-HPSD and placebo conditions. Importantly, post-exercise blood glucose levels were significantly elevated following the consumption of B-HPSD (52) (**Table 4**). Dora et al. examined the impact of essential amino acid (EAA) supplementation, predominantly consisting of BCAAs, on cognitive performance and circulating levels of BDNF following moderate-intensity aerobic exercise. A cohort of twenty-two healthy young males consumed either EAAs or a placebo 30 minutes prior to engaging in cycling at 60% of their peak oxygen uptake for a duration of 30 minutes. Notably, executive function (EF) exhibited a significant enhancement post-exercise in the EAA group in comparison to the placebo group, while memory recognition (MR) and circulating BDNF concentrations remained unchanged. The observed improvement in EF was positively correlated with elevated levels of specific amino acids—namely leucine, isoleucine, valine, lysine, and phenylalanine—which function as precursors for neurotransmitter biosynthesis within the central nervous system. However, these findings were obtained in healthy individuals under acute exercise conditions and therefore should not be directly interpreted as evidence for therapeutic cognitive improvement in depressive disorders (53). Caldo-Silva et al. analyzed the combined impacts of ME and BCAAs supplementation on inflammatory biomarkers, serum albumin, and both physical and cognitive performance in a cohort of frail elderly individuals (≥75 years) residing in assisted living facilities. Participants were systematically allocated to one of four groups: ME + BCAAs, ME exclusively, BCAAs exclusively, or a control cohort, throughout a 40-week multifactorial intervention that included washout intervals. Plasma levels of albumin, pro- and anti-inflammatory cytokines (TNF-α, IL-10), the TNF-α/IL-10 ratio, and myeloperoxidase (MPO) activity were quantified, alongside assessments of muscular strength, cognitive functioning, and physical frailty. The ME + BCAAs group exhibited enhancements in cognitive performance and muscle-strength-related albumin concentrations, coupled with diminished TNF-α levels; however, no statistically significant alterations were detected in the TNF-α/IL-10 ratio or MPO activity. Although improvements in cognitive measures were observed, the multifactorial design involving both exercise and nutritional intervention limits the ability to isolate the specific contribution of BCAAs (54).

**Table 4:** Indirect mechanistic and cognition-related evidence regarding BCAA supplementation combined with exercise.

Participants/model	Intervention	Duration	Main outcomes	Findings	Evidence category	Study
11 experienced cyclists	B-HPSD (2,256 mg BCAA + 300 mcg VitB-6) vs. PLA	Acute, single session	Multiple sprint exercise (MSE) performance, blood glucose, lactate, HR, RPE, cognitive function	MSE performance →; post-exercise blood glucose ↑; lactate, HR, RPE →; cognitive performance →	Exercise/cognition-related evidence	(52)
22 healthy young men	EAA supplements (mainly BCAA) vs. PLA	Single session, 30 min aerobic exercise at 60% VO_2_peak	Executive function (EF), memory recognition (MR), circulating BDNF	EF ↑; MR →; BDNF →	Indirect mechanistic evidence	(53)
Frail older persons (≥75 y; RCH residents; n=35)	ME + BCAA, ME, BCAA, control	40 weeks (16 wk intervention – 8 wk washout – 16 wk intervention)	Albumin, inflammatory markers (IL-10, TNF-α), TNF-α/IL-10 ratio, MPO, muscle strength, cognitive profile	Cognitive profile ↑; muscle strength ↑; albumin ↑; TNF-α ↓; TNF-α/IL-10 ratio →; MPO →	Indirect clinical/mechanistic evidence	(54)
Endurance athletes (30-km cross-country runners)	BCAA in carbohydrate solution vs. placebo	Single 30-km run	Mood, cognitive performance (color-word test, shape-rotation, figure-identification tasks)	Complex cognitive tasks ↑; less demanding tasks →; mood →	Exercise/cognition-related evidence	(55)
Ldlr-/-.Leiden mice (HFD-induced obese)	Exercise, Exercise + BCAA, no exercise	6 months	Brain structure, neuroinflammation, cerebral blood flow, cognition, metabolic parameters	Exercise alone: cerebral blood flow ↑; white matter loss ↓; neuroinflammation ↓; cognition →; Exercise + BCAA: cerebral blood flow →; white matter loss →; neuroinflammation ↑; cognition ↑ slightly; metabolism: epididymal fat %, muscle weight ↑; body weight ↓; insulin ↓; grip strength ↑ transiently	Indirect mechanistic evidence	(56)

B-HPSD, BioSteel High Performance Sports Drink; BCAA, branched-chain amino acids; VitB-6, vitamin B-6; PLA, placebo; MSE, multiple sprint exercise; PO, power output; HR, heart rate; RPE, ratings of perceived exertion; EAA, essential amino acids; EF, executive function; MR, memory recognition; BDNF, brain-derived neurotrophic factor; ME, multicomponent exercise; PF, physical frailty; RCH, residential care homes; IL-10, interleukin-10; TNF-α, tumor necrosis factor-alpha; MPO, myeloperoxidase.

**Figure 1:**
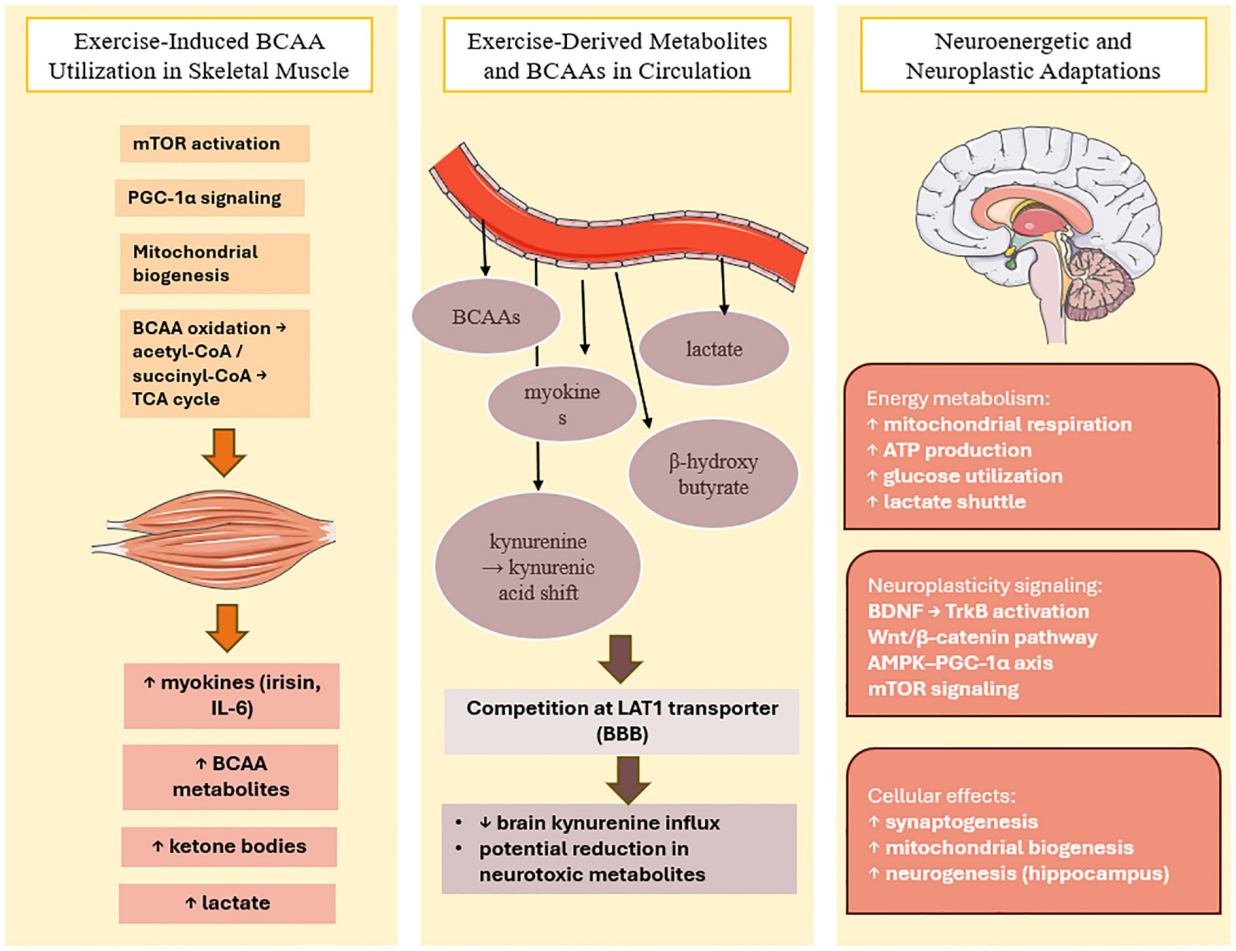
Proposed mechanistic framework linking branched-chain amino acid (BCAA) supplementation and exercise to improvements in cognitive function. Exercise increases skeletal muscle oxidation of BCAAs and stimulates the release of metabolic intermediates and myokines into circulation. Circulating BCAAs and exercise-derived metabolites influence transport processes at the blood–brain barrier and alter central metabolic signaling. Within the brain, these signals may enhance mitochondrial function, activate neuroplasticity pathways (BDNF–TrkB, Wnt/β-catenin, AMPK–PGC-1α), and support hippocampal neurogenesis. These adaptations may contribute to improvements in executive function, working memory, and cognitive flexibility—domains frequently impaired in MDD. This evidence is drawn from a relatively limited number of studies using small samples, with very particular participant groups, such as experienced cyclists, healthy young males, and elderly people who were already frail, thus reducing its external validity. Not only this, but there was considerable variability across the studies in the specifics of their intervention protocols regarding their composition of amino acids, timing of delivery, type of physical activity, as well as its intensity and duration. Some of these studies examined the impact of the supplements in the short term, using measures of acute cognitive performance, which might be an inadequate measure of their neurological effects. In addition, the inability to blind participants to the intervention protocol in some of these studies, along with insufficient risk of bias assessments, allocation concealment, and reporting of dropouts, further undermines confidence in the evidence.

Another research examined the impact of BCAAs supplementation on central fatigue and cognitive function during a 30-km cross-country race. Within exercise physiology, prolonged physical exertion may increase central serotonergic activity in brain regions involved in fatigue perception. Under these conditions, BCAAs may attenuate exercise-induced central fatigue by lowering the plasma free tryptophan-to-LNAAs ratio and thereby reducing acute increases in central 5-HT synthesis. Importantly, these acute neurochemical responses occur within the context of exercise performance and should not be considered mechanistically equivalent to serotonergic dysfunction in MDD. Importantly, the reduction of exercise-induced central fatigue through modulation of serotonergic activity should not be interpreted as equivalent to improving serotonergic dysfunction in clinical depression. The participants were administered either a BCAAs-carbohydrate mixture or a carbohydrate-only placebo for comparative analysis. Cognitive performance was evaluated both prior to and subsequent to the race through the implementation of color-word, shape-rotation, and figure-identification tasks. The supplementation of BCAAs yielded enhancements in performance on complex tasks such as color-word tests by 3–7% and maintained performance levels in shape-rotation and figure-identification tasks; conversely, participants receiving the placebo exhibited declines in performance ranging from 15% to 25%. BCAAs exerted negligible influence on less complex tasks and exhibited slight modulation of mood alterations induced by exercise. These findings primarily reflect preservation of cognitive performance under prolonged physical stress conditions rather than direct antidepressant or cognition-restorative effects in depressive pathology (55). Lohkamp et al. examined the implications of physical exercise and BCAAs supplementation on cerebral function and metabolic processes in Ldlr-/- Leiden mice subjected to a high-fat diet. The implementation of exercise in isolation resulted in enhanced cerebral perfusion, diminished white matter degeneration, and a reduction in neuroinflammatory responses, likely attributable to augmented vascular and glial support mechanisms. When BCAAs supplementation was integrated, a notable increase in neuroinflammation was observed, notwithstanding marginal enhancements in cognitive performance, which suggests the potential pro-inflammatory consequences of excessive amino acids on glial or microglial activation pathways. The combination of BCAAs and exercise led to an elevation in muscle mass and epididymal adipose tissue, concomitantly decreasing body weight and levels of fasting insulin, thereby indicating improved protein synthesis alongside enhanced insulin signaling mechanisms. From a mechanistic perspective, exercise likely facilitated modulation of neurovascular coupling, neurotrophic signaling, and oxidative stress responses, while BCAAs appeared to influence amino acid metabolism and the availability of neurotransmitter precursors. The findings shown that BCAAs supplementation in conjunction with exercise can have divergent effects on metabolic, inflammatory, and neurocognitive pathways, thus necessitating a judicious approach in the application of such interventions aimed at addressing obesity-related cognitive dysfunction (56) (**Figure 1**). Importantly, the observation that BCAAs supplementation may simultaneously improve certain metabolic parameters while increasing neuroinflammatory markers further highlights the complexity of extrapolating these findings to depression-related cognitive dysfunction. Furthermore, the divergent metabolic and neuroinflammatory effects observed in this animal model underscore that BCAA-related cognitive outcomes are highly context-dependent and may vary according to physiological state, dietary background, and exercise conditions. Therefore, caution is warranted when extrapolating these findings to human depressive disorders. Overall, the available evidence suggests that BCAAs may influence cognitive performance primarily through mechanisms related to exercise physiology, fatigue resistance, metabolic adaptation, and maintenance of substrate availability during periods of physical stress. However, these findings should not be overgeneralized to imply direct therapeutic efficacy for cognitive impairment associated with MDD. Importantly, the neurobiological mechanisms underlying cognitive dysfunction in depression extend beyond alterations in amino acid metabolism and include chronic disturbances in affective processing, neuroinflammation, stress-related neural remodeling, and dysregulation of limbic-cortical networks. Consequently, preservation of cognitive performance during exercise or acute physiological stress in healthy individuals cannot be equated with cognitive remediation in depressive disorders. Most studies reviewed in this section were conducted in healthy, athletic, aging, or animal populations under acute experimental conditions, and direct clinical evidence examining the combined effects of BCAAs and exercise on cognition in patients with MDD is currently scarce. Therefore, within the conceptual framework proposed in this review, BCAAs should primarily be regarded as modulators of exercise capacity and metabolic resilience, with any potential cognitive or mood-related benefits occurring indirectly through enhanced physical activity and subsequent neuroplastic adaptations rather than through direct antidepressant mechanisms.

## 7 Future perspectives and clinical implications

Despite the increasing mechanistic interest in creatine supplementation, BCAAs, and physical exercise in pathways related to depression and cognitive function, the current evidence base remains insufficient to support any clinical application in MDD. Therefore, the interaction between these interventions should be considered a theoretical and hypothesis-driven framework rather than an established therapeutic strategy. Future research should prioritize large-scale, well-designed randomized controlled trials to determine whether the proposed interactions translate into meaningful clinical effects in patients with MDD. At present, most available evidence is derived from preclinical models or studies conducted in healthy or non-depressed populations, limiting direct clinical interpretation. From a translational perspective, future investigations should focus on clarifying mechanistic interactions among metabolic regulation, neurotransmitter dynamics, and neuroplasticity. Importantly, future studies should explicitly distinguish between exercise-related serotonergic responses and depression-related serotonergic dysfunction, as these represent biologically distinct phenomena operating on different temporal and neuroanatomical scales. Acute alterations in tryptophan availability and central fatigue mechanisms during exercise should not be assumed to reflect the chronic serotonergic abnormalities characteristic of MDD. Accordingly, future experimental designs should incorporate biomarkers capable of differentiating these parallel processes, including assessments of exercise-related amino acid dynamics, raphe nuclei function, receptor regulation, and neuroplasticity-associated signaling pathways. However, these mechanisms should be interpreted cautiously, as current findings remain largely indirect and context-dependent. Importantly, individual variability (e.g., sex, age, genetic background, nutritional status, and baseline physical activity) should also be considered when designing future studies, as these factors may influence metabolic and neurobiological responses to these interventions. In addition, longitudinal clinical studies conducted specifically in patients with MDD are required to determine whether improvements in exercise capacity, fatigue resistance, and brain bioenergetics ultimately translate into clinically meaningful antidepressant and cognitive benefits.

## 8 Recommendations for future preclinical and clinical research

Despite growing interest in the potential interaction between creatine supplementation, BCAAs, and exercise in regulating brain function, mood, and cognition, the current literature remains limited by methodological heterogeneity and a lack of translational validation. Accordingly, future research should aim to systematically test the validity of the proposed mechanistic model rather than assume its clinical relevance. From a preclinical perspective, studies should further investigate whether combined or independent effects of creatine, BCAAs, and exercise converge on shared pathways related to brain energy metabolism, mitochondrial function, neurotransmitter regulation, and neuroplasticity. However, it remains unclear whether these mechanistic findings observed in experimental models are transferable to depressive pathology in humans. Future studies should also better isolate the independent versus combined contributions of each intervention component, as many existing studies use multi-ingredient designs that limit causal interpretation. Future preclinical studies should also explicitly distinguish between exercise-related serotonergic adaptations and depression-related serotonergic dysfunction. In particular, experimental models should determine whether alterations in plasma tryptophan/BCAA competition and acute central fatigue mechanisms are mechanistically independent from the chronic serotonergic abnormalities observed in depressive states. Such investigations may help clarify whether these represent parallel but biologically distinct processes rather than different manifestations of a single serotonergic pathway. Dose–response relationships, timing of supplementation relative to exercise, and long-term adaptations should also be systematically explored. From a clinical perspective, there is a need for adequately powered randomized controlled trials involving clearly defined depressive populations. These studies should adopt standardized protocols for both supplementation and exercise interventions, along with harmonized outcome measures. Importantly, future clinical trials should incorporate biomarkers capable of differentiating acute exercise-induced serotonergic responses from chronic depression-related serotonergic dysfunction. Potential biomarkers may include assessments of amino acid availability, exercise-related fatigue indices, neuroplasticity markers (e.g., BDNF), and measures of central serotonergic function and limbic-cortical network activity. In addition, future research should not only aim to evaluate outcomes but also to determine whether the proposed creatine–BCAA–exercise framework is biologically and clinically valid in the context of MDD. Particular attention should be paid to establishing whether improvements in exercise capacity and reductions in central fatigue translate into clinically meaningful antidepressant effects through indirect neuroplastic mechanisms rather than direct serotonergic modulation.

## 9 Limitations

Several important limitations should be considered when interpreting the current literature on creatine supplementation, BCAAs, and exercise in relation to depression-related outcomes. A substantial proportion of the available evidence is derived from studies conducted in healthy individuals, athletes, older adults, sleep-deprived participants, or animal models rather than clinically diagnosed populations with MDD. Many of these studies primarily assess exercise performance, fatigue, cognition, or metabolic responses, rather than depressive pathology itself. Consequently, while these findings provide valuable mechanistic insights, they should be interpreted as indirect and non-clinical evidence. In addition, the interpretation of BCAA-related serotonergic mechanisms should be approached cautiously. Although the “central fatigue hypothesis” describes well-established metabolic competition between BCAAs and tryptophan at the blood–brain barrier, these mechanisms originate from exercise physiology and do not directly reflect serotonergic dysfunction in MDD. Importantly, exercise-related serotonergic responses and depression-related serotonergic dysfunction represent biologically distinct phenomena that differ in their neuroanatomical substrates, temporal characteristics, and functional significance. Consequently, acute changes in tryptophan transport and central fatigue mechanisms during exercise should not be assumed to reflect chronic alterations in raphe nuclei activity, receptor sensitivity, or limbic-cortical serotonergic signaling observed in MDD. Therefore, such findings should not be interpreted as evidence of antidepressant effects. Rather, the potential contribution of BCAAs to depression-related outcomes should currently be regarded as indirect and primarily mediated through their influence on exercise capacity, fatigue perception, and associated neuroplastic adaptations. The current literature is also highly heterogeneous in terms of study design, intervention duration, dosing strategies, exercise protocols, and outcome measures. Many studies suffer from small sample sizes and short intervention periods, limiting statistical power and generalizability. Furthermore, a significant portion of mechanistic evidence originates from preclinical animal studies, which, although valuable for understanding biological pathways, cannot fully replicate the complexity of human depressive disorders. Taken together, the current evidence base should be considered preliminary and exploratory, supporting a conceptual model rather than a validated clinical framework.

## 10 Conclusion

Depression is a multifactorial neuropsychiatric disorder characterized by dysregulation of neurotransmitter systems, impaired cerebral energy metabolism, and altered neuroplasticity. Within this context, creatine supplementation, BCAAs, and physical exercise have been investigated as components of a hypothetical mechanistic framework linking brain energy metabolism, amino acid transport, and neuroplastic processes. Creatine may contribute to neuronal bioenergetics through the phosphocreatine system, while BCAAs may influence amino acid transport dynamics at the blood–brain barrier and indirectly modulate serotonergic availability under metabolic conditions. Importantly, the serotonergic effects of BCAAs appear to be primarily relevant to exercise-related central fatigue mechanisms and should not be considered mechanistically equivalent to the chronic serotonergic dysfunction observed in MDD. Acute exercise-induced alterations in tryptophan transport and serotonin availability represent physiological adaptations that are biologically distinct from depression-related abnormalities involving raphe nuclei activity, receptor sensitivity, and limbic-cortical circuitry. Exercise is well established to promote neuroplasticity and neurotrophic signaling, including BDNF-related pathways. However, it is critical to emphasize that most current evidence is derived from preclinical studies or non-depressed populations. Therefore, the proposed interactions should be interpreted as indirect mechanistic associations rather than clinically validated effects in MDD. Accordingly, the potential contribution of BCAAs to depression-related outcomes is likely indirect and mediated predominantly through improvements in exercise capacity, fatigue resistance, and associated neuroplastic adaptations rather than through direct serotonergic antidepressant effects. Overall, the creatine–BCAA–exercise framework should currently be viewed as a hypothesis requiring rigorous validation in well-designed translational and clinical studies, rather than an established strategy for improving cognitive or emotional outcomes in depression. Future research is required to determine whether this mechanistic model has true clinical relevance in depressive disorders.
